# Expression-Based Network Biology Identifies Alteration in Key Regulatory Pathways of Type 2 Diabetes and Associated Risk/Complications

**DOI:** 10.1371/journal.pone.0008100

**Published:** 2009-12-07

**Authors:** Urmi Sengupta, Sanchaita Ukil, Nevenka Dimitrova, Shipra Agrawal

**Affiliations:** 1 Institute of Bioinformatics and Applied Biotechnology, Bangalore, Karnataka, India; 2 BioCOS Life Sciences Private Limited, Bangalore, Karnataka, India; 3 Philips Research North America, Briarcliff Manor, New York, United States of America; University of Maryland, United States of America

## Abstract

Type 2 diabetes mellitus (T2D) is a multifactorial and genetically heterogeneous disease which leads to impaired glucose homeostasis and insulin resistance. The advanced form of disease causes acute cardiovascular, renal, neurological and microvascular complications. Thus there is a constant need to discover new and efficient treatment against the disease by seeking to uncover various novel alternate signalling mechanisms that can lead to diabetes and its associated complications. The present study allows detection of molecular targets by unravelling their role in altered biological pathways during diabetes and its associated risk factors and complications. We have used an integrated functional networks concept by merging co-expression network and interaction network to detect the transcriptionally altered pathways and regulations involved in the disease. Our analysis reports four novel significant networks which could lead to the development of diabetes and other associated dysfunctions. (a) The first network illustrates the up regulation of *TGFBRII* facilitating oxidative stress and causing the expression of early transcription genes via MAPK pathway leading to cardiovascular and kidney related complications. (b) The second network demonstrates novel interactions between *GAPDH* and inflammatory and proliferation candidate genes i.e., *SUMO4* and *EGFR* indicating a new link between obesity and diabetes. (c) The third network portrays unique interactions *PTPN1* with *EGFR* and *CAV1* which could lead to an impaired vascular function in diabetic nephropathy condition. (d) Lastly, from our fourth network we have inferred that the interaction of β-catenin with *CDH5* and *TGFBR1* through Smad molecules could contribute to endothelial dysfunction. A probability of emergence of kidney complication might be suggested in T2D condition. An experimental investigation on this aspect may further provide more decisive observation in drug target identification and better understanding of the pathophysiology of T2D and its complications.

## Introduction

Diabetes is a serious health problem in society, and about 90% of the diabetic population is affected with T2D [1]. According to the International Diabetes Federation (IDF) approximately 246 million adults in the seven IDF countries were living with T2D in 2007. This number is expected to increase to 380 million by 2025 (IDF, http://www.idf.org/). The disease is characterized by impaired glucose homeostasis, decreased insulin activity and insulin resistance which lead to elevated blood glucose levels [2], [3]. The advanced form of the disease causes acute cardiovascular, renal, neurological and organ complications [4]–[8].

This metabolic condition is determined by the interaction of various environmental and genetic factors. Obesity is a major risk factor in T2D development [9]. Elevated levels of free fatty acids (FFA) in obesity promote interactions between FFA, lipid metabolites, inflammatory pathways and mitochondrial dysfunction [10]–[12]. Research investigations to unravel the molecular mechanism of T2D have led to the identification of multiple signalling and metabolic pathways that get altered during the disease. Insulin resistance is the main underlying cause of several transcriptionally altered signalling and metabolic pathways in T2D which later lead to defective microvascular, macrovascular and endothelial functions [13]. Thus far, alteration in signalling pathways mediated by insulin, adipocytokines, FFA, *EGF*, Jak/STAT, *MAPK*, *VEGF*, *PPAR*, *PI3-K* and *Wnt* have been reported in the pathogenesis of T2D. *EGF* exerts insulin like effects on glucose transport and lipolysis and can increase the tyrosine phosphorylation and activation of *IRS-1* and *IRS-2*. *EGF* is also capable of activating additional *PI3-K* pools and, thereby augments the downstream signalling of insulin in insulin-resistant states like T2D [14]. It has been found that high glucose concentration causes production of *TGFB* and activates Jak/STAT signalling cascade in diabetic kidney cells. Activation of this signalling cascade can stimulate excessive proliferation and growth of glomerular mesangial cells, contributing to diabetic nephropathy [15], [16]. Exposure to high glucose concentration has also been shown to activate MAPK signalling pathway in skeletal muscle cells [17]. Impairment in VEGF signalling has been noticed in T2D. Chronic coronary heart disease in diabetic patients is characterized by an increased *VEGF* myocardial expression and a decreased expression of its receptors along with down-regulation of its signal transduction resulting in reduced neoangiogenesis [18]. Signalling pathway mediated by *PPAR* is down-regulated in diabetes [19]. Mitogenic stimulation mediated by MAPK signalling cascade suppresses *PPARG* activity [20]. *PI3-K* is a key molecule in insulin signalling which is found to be down-regulated in T2D [21]. Wnt signalling process plays an important role in pancreatic beta-cell development by promoting expression of Pitx2 and CyclinD2 which regulate beta cell cycle progression [22]. Reactive oxygen species (ROS) production by FFA has also been implicated in pancreatic cell death. ROS activates *NF-kB* which eventually leads to apoptosis and/or necrosis of beta cells [23]. Thus it is seen that attenuation in insulin signalling seems to affect/induce cross-talk among various processes responsible for apoptosis, endothelial dysfunction and vascular dysfunction [24], [25]. Other than these pathways, a number of genes have been discovered to be candidates to cause T2D.

The aim of this study is to put forth novel biological networks that describe transcriptional alteration (up and/or down-regulation) in genes/pathways which could contribute to the pathogenesis of T2D and its associated complications. Knowledge and statistics based systematic analysis of high throughput molecular data from normal and diseased individuals can be used to construct candidate molecular networks. An extensive analysis of these networks facilitates the identification of pathways and genes affected during the disease process. Similar approach is comprehensively being used to identify candidate genes and biomarkers for various complex diseases including cancers and diabetes [26]–[29]. Bergholdt et al [30], identified loci showing genetic interactions associated with Type 1 Diabetes (T1D) using genome scan data. By elucidating possible epistasis between classic T1D loci, major T1D predictive signals (marginal markers) were characterized and fine mapped. In order to elucidate/identify underlying biological interactions and novel candidate genes, the genetic epistasis analysis data were integrated with protein networks spanning the interacting epistatic regions and scanned for functional sub networks.

We have applied a network biology approach which involves the integration of co-expressed gene network with corresponding protein interaction network to identify signature networks. In our study, we have worked with microarray data comprising diabetic and other complications. Instead of selecting genes from susceptible diabetic risk loci, we have considered all those genes that appeared differentially expressed in our analysis. The genetic networks were integrated with corresponding protein interaction networks. Integration of independent but biologically related genetic, molecular and regulatory information appears as a reliable method to obtain insights into functional modules which allow detection of previously unknown deregulated pathways [31]–[33]. Through this approach we tried to assess the interactions of known T2D candidate genes with other molecules in different biological pathways and a few unique interactions which could result in new, non-obvious hypotheses that are statistically significant.

In the work we present here, microarray gene expression data analysis identifies transcriptionally altered key genes involved in signalling/metabolic pathways of T2D. In addition, the protein-protein interaction data enables understanding of the protein complexes and their molecular organization in the overall topology of the networks. The combined analysis of expression profiles and protein-protein interaction data in integrated networks have been shown to generate significant molecular mechanisms and pathways. Our results depict their potential involvement in diabetes progression and various associated complications as well.

In the current study we have computationally constructed four new sub networks and on analyzing these networks, we have proposed different possible alterations of signalling pathways in these networks. We have predicted novel molecular regulators (unique genes and interactions) which could have an impact on the pathophysiology of T2D and its complications via various significant pathways such as insulin signalling, oxidative metabolism, Wnt signalling and others. The present system level network biology analysis from diabetes and obesity microarray expression datasets shows that the interaction across *TGFBRII, SMAD3* and *GCR* along with FFA can induce vascular complications in diabetes. It is suggested from the study that *GAPDH*, a significant enzyme in carbohydrate metabolism, can induce micro vascular complications and faulty insulin signalling in association with *SUMO4*, growth factor *EGFR* and *IRS* in diabetic and obese individuals. A careful modular dissection and examination of networks from diabetic nephropathy datasets exhibit the interactions between *EGFR* with *PTPN1* and *CAV1* through *AKT1* activation. Analyses of the three large transcriptionally altered diabetic datasets (Mexican_Hs, IR_Hs and DN_Hs) demonstrate that the oxidative stress induced deregulation of *β-catenin* plays an important role in causing kidney diabetic complications by the down-regulation of *CDH5*.

## Materials and Methods

### Details of Microarray Datasets

Seven studies from human and one study from mouse encompassing a set of 138 microarray expressions from human and 12 from mouse have been selectively retrieved from GEO (Gene Expression Omnibus) datasets and Diabetes Genome Anatomy Project for the present analysis. The selection of microarray expression studies is based on the following criteria: (i) studies that examine the insulin resistance associated with T2D pathogenesis (ii) nephropathy as one of the major T2D associated complications (iii) obesity contributing to insulin resistance via the development of cardiovascular disease and (iv) inflammation caused by obesity. All the datasets have been named according to the study type as prefix and species as suffix (Studytype_Species), ‘Hs’ indicating human dataset and ‘Mm’ indicating mouse dataset. All the datasets along with their tissue source and number of control and diseased samples have been detailed in Dataset S1. Aiming to address different altered biological pathways and gene/protein interactions in T2D, we focused on those datasets which could be more related to the problems associated with this disease.

Dataset IR_Hs originally reports that the differentially expressed genes in insulin resistance of skeletal muscle cells are susceptible genes for T2D [34]. The datasets Preadipocyte_Hs and Adipocyte_Hs describes obesity induced inflammatory response in preadipocytes and adipocytes cells [35], [36]. Another dataset, Obs_Hs, reports differentially expressed obesity responsive genes, which further relates to T2D pathophysiology through insulin resistance [37]. Expression analysis from PCOS_Hs dataset shows that the obesity can cause polycystic ovary syndrome, which is independent of insulin resistance in women [38]. Kidneys are highly affected in acute diabetic condition. DN_Hs and Renalfailure_Mm describe diabetic nephropathy in the kidney tissue and loss of its damage repair capability [39], [40]. Mexican_Hs dataset reports differentially expressed genes specific to insulin resistance and T2D [41].

### Data Preparation

**Figure 1:**
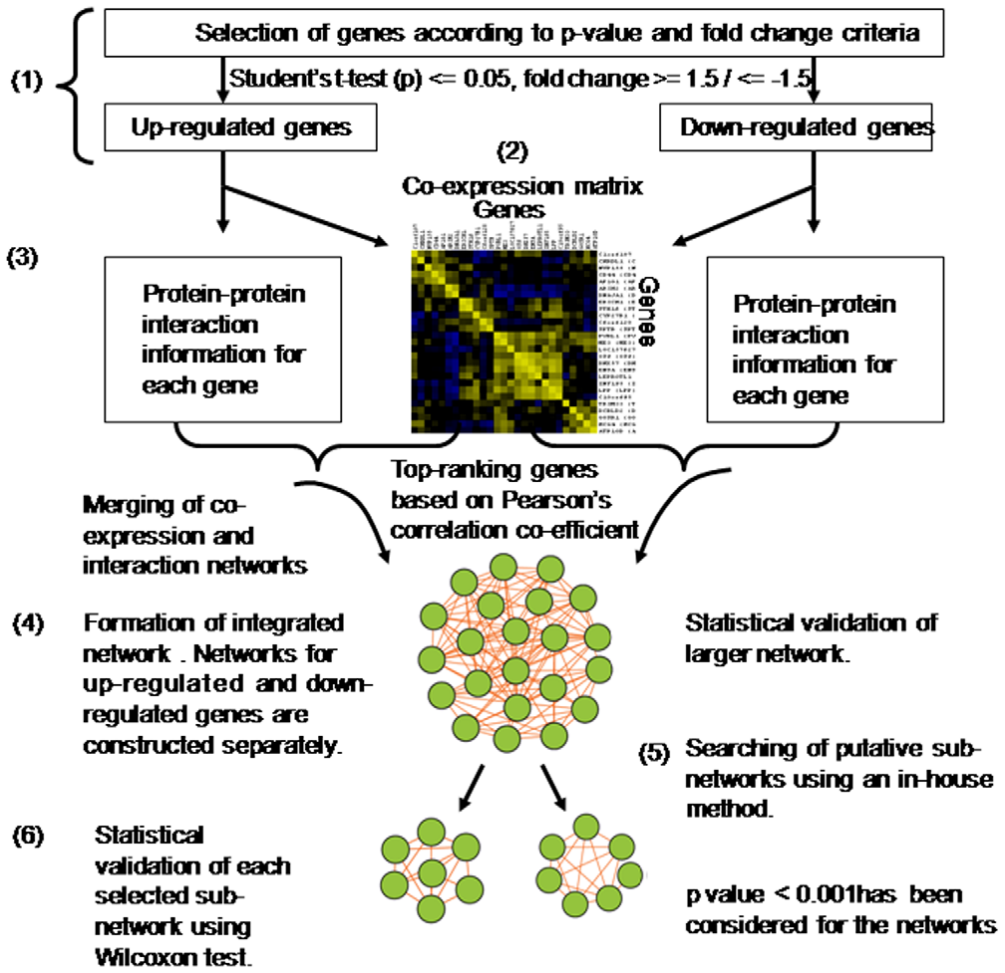
Overview of integrated network of gene-gene co-expression and protein-protein interaction information of microarray data (1-6). (**1**) All the datasets have been filtered through the defined cut-off of student's t-test and fold change value. The differentially expressed up and down-regulated genes were sorted to make separate lists. (**2**) The co-expression network was constructed by calculating Pearson's correlation coefficient (r-value) for each gene pair. And the top-ranking genes (r-value varies for the datasets) were used to construct the co-expression network. The corresponding gene sets were processed to generate the protein-protein interaction networks. (**3**) **&** (**4**) Both the networks were merged to get the final large network. The statistical validation of the large network was done using Wilcoxon test. (**5**) The potential sub-networks were identified using an in-house Java program. (**6**) Sub-networks were further validated through Wilcoxon test. The other validation parameters like average clustering coefficient, average degree and network density have also been calculated to assess the statistical significance of the network.

Each dataset is normalized in order to bring the unit variance across the data using the following two steps: (a) Calculation of row-wise mean and standard deviation for each gene in all the data files. (b) Subtraction of mean from each expression value (from both control and diseased sets) followed by the division of the resultant value by standard deviation. This is done across all the datasets. Figure 1 illustrates the overall method.

### Selection of Genes

Each dataset varies in terms of experimental conditions, types and numbers of samples and the number of differentially expressed genes. Therefore, identification of the significant genes from each dataset is a crucial step in the process of analysis. DNA Chip Analyzer [42] has been used for comparing the microarray datasets (diseased vs. controls). Differentially expressed genes are identified from the normalized datasets at the cut-off p-value < = 0.05 and fold change value > = 1.5 and < = −1.5. Genes satisfying these conditions are grouped separately as up-regulated and down-regulated genes (Dataset S2).

### Construction of Protein-Protein Interaction Network

Protein-protein interaction network for each set of up and down-regulated genes has been constructed by APID2NET, an implemented plug-in of Cytoscape [43]. APID2NET retrieves all the possible information on protein-protein interaction from five interaction databases namely, Database of Interacting Proteins [44], Biomolecular Interaction Network Database [45], IntAct [46], Molecular Interactions Database [47] and Human Protein Reference Database [48]. Swissprot/Uniprot IDs for each group of genes have been collected from APID database [49] and then imported to Cytoscape via its plug-in. The up-regulated and down-regulated genes from each study were separately submitted in APID2NET and the networks were analyzed in Cytoscape platform.

### Construction of Co-Expression Network

The expression similarity across the gene datasets was derived using Pearson's correlation coefficient (r-value). A Pearson's correlation coefficient gives the measurement of the degree of the correlation between two variables. Cladist software was used to construct correlation matrix based on r-values for each dataset [50]. An in house java program was written to rank the gene-pairs on the basis of their correlation values. It generates a symmetrical n x n matrix at a given r cut-off value (range: 0.6–0.9). The rank matrix was imported in Cytoscape to construct the network of co-expressed genes.

### Construction of Integrated Gene Networks Using Expression and Interaction Data

In network organization each gene or protein is represented as a **node**. The number of interactions or links that a node has with other nodes is defined as a **degree**. Co-expression network comprises nodes which correspond to genes and the edges corresponding to co-expression links. The protein-protein interaction network obtained from APID includes information on co-interacting proteins, defined as proteins that have physical interaction. APID provides known experimentally validated protein-protein interactions. The edges in the integrated functional network correspond to both co-expression and physical protein-protein interactions.

The approach underlying the present study utilizes an integrated concept of merging the gene-gene co-expression correlation matrix and protein-protein interaction data to construct the complete networks (eight studies). Integration refers to the process of combining networks by merging nodes that share a particular GO annotation, or nodes whose gene expression levels change significantly in one or more conditions according to p-values loaded with the gene expression data. Integrated networks were created by overlapping nodes that were common to both co-expression and protein interaction networks. Both co-expression networks and protein interaction networks were integrated using Cytoscape plug-in which identifies the genes by their ID types. These networks have many embedded sub-networks with significant biological functions relevant to T2D pathogenesis.

### Searching of High-Scoring Sub-Networks

A sub-network of large protein-protein interaction network can be defined as the set of statistically and functionally significant interacting genes. The potential sub-networks have been identified by a search method estimating their significance scores [30]. The significance score (S) is calculated as, S = average (s_1_, s_2_,…, s_n_), where ‘s’ is the individual node score. ‘s’ for each node is computed by dividing the total number of direct interactions (i.e., first order neighbors) of that node by the average degree present in the sub-network. A cut-off value of 0.5 is set to consider those nodes which have significant number of interactions in the network. Nodes with higher than 0.5‘s’ value have been taken for further search. All the individual node scores have been averaged to get the final ‘S’ score. The threshold value for ‘S’ score has been set as 1 and sub-network showing higher than this value has been taken into consideration. The four sub-networks showing high scores have analyzed in the succeeding sections. Thus the sub networks were extracted from the larger networks generated by integrating expression data and interaction data. They were further assessed statistically and analyzed using the information available from literature sources and online repositories to verify their biological functions pertaining to T2D.

### Evaluation of Topological and Statistical Measures of the Sub-Networks

The topological and statistical significance of each sub-network, abstracted from large networks have been calculated using Cytoscape plugins Network Analyzer [51] and CentiScaPe [52]. We have used the following network biology concepts to evaluate the topology and extent of clustering in the candidate sub-networks:

Topological coefficient for node n_1_ is computed as TC (n_1_) = average (J(n_1_, n_2_)/k_n1_), where J(n_1_, n_2_) gives the value of the number of nodes shared by both n_1_ and n_2_ nodes and k_n1_ is the number of interactions of node n_1._

Average clustering coefficient measures the average of clustering coefficients of all nodes, which are defined as the ratio of the number of edges between the neighbours of a node to the maximum number of edges that could possibly exist between them. It can be expressed as C (n) = 2e_n_/(k_n_(k_n_-1)), where k_n_ is the no. of interactions of node n and e_n_ is the no. of connected pairs between all neighbours of node n. Log-log graphs have been constructed by plotting the number of neighbours “k” on x-axis and the average clustering coefficient “C(k)” and topological coefficient “TC(k)” as the functions of k on y-axis.

Average degree measures the average of all connectivities of a node. This is extended by network density, which indicates the compactness of one network distributed through its edges. The results were further refined for all the sub-networks by estimating Wilcoxon test in R package.

Usually centrality measures are used to capture the structure of node in the network and identify the hub proteins. The simplest measure of all centralities is node degree distribution. The degree of a node v is the number of nodes that are directly connected to it i.e. first neighbours of node v. We calculated the degree distribution P (k), which determines the probability of node v with k number of links, where k = 1, 2…This pattern of structure obeys the power law P(k) ∼ k^−γ^, where γ is a constant called degree exponent indicating the scale-freeness of networks. By fitting a line to the given sets of data the pattern of their dependencies can be seen which can be used to validate the scale-free topology of the networks. The software used here uses least square method [53] and considers only the data points with positive co-ordinate values for fitting the line, where the power-law curve is *y* = β *x* ^α^. This model is transformed into ln *y* = ln *β*+*α* ln *x*. When a plot is made and the coefficient of determination, R^2^ of the regression line is computed, the network models can be tested based on this value [54]. The R-squared value measures how well the data points fit to the curve.

Another centrality measure, the betweenness centrality calculates a value of a node (n_1_) that is located in the shortest path of two other nodes (n_2_ and n_3_) and indicates its significance in the communication of these two nodes [55]. The betweenness value of n_1_ can be expressed as,
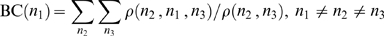



Here ρ (n_2_, n_3_) is the number of shortest paths starting at node n_2_ and ending at node n_3_. The value of ρ (n_2_, n_1_, n_3_) indicates the number of those shortest paths which pass through node n_1_ in the network.

### Scale-Freeness Topology of the Networks

Biological networks have been characterized by topological features which establish their scale-freeness property [56]. Protein interaction networks and co-expression networks also exhibit a scale-free geometry, where the nodes are not uniformly populated with neighbours. All the nodes of these networks do not follow the rule of having an average number of links per node. Most of the nodes have few partners, while a few nodes also called ‘hubs’ interact with many partners [26]. Power law process is used for estimating the parameters and validating the network models with their scale-freeness property. Usually R-squared values closer to 1 indicate higher correlation and a stronger linear relationship between the data variables. Here also the R-squared values obey the rule emphasising that the networks are scale-free i.e., they are unevenly populated with hubs and less dense nodes. Biological networks are found to be very sensitive to the removal of hub proteins. It has been observed that the deletion of hub proteins in yeast protein-protein interaction network exerts an increased lethal effect [57]. In the present study is has been observed that the hub proteins are communicating with many other significant proteins involved in many pathways reported to be affected during T2D. Further biochemical investigation on the removal of these hub proteins needs to be conducted to provide better understanding in the roles played by them in the pathophysiology of T2D.

### Analysis of Functional Enrichment of the Networks

In order to identify how the networks are functionally embellished we used GOlorize, a Cytoscape plug-in [58]. It is based on a hyper geometric test with Benjamini and Hochberg false discovery rate (FDR) corrected p-value and displays the overrepresented functional gene ontology (GO) categories in a given network. The major functional categories have been taken to construct pie diagrams based on their overall frequencies in a network.

### Validation of Novel Interactions

Two new interactions observed in the networks were validated by the identification of their interacting domains. InterDom [59], [60] was used to predict the interacting domains for each protein pair and further verified using 3did [61].

## Results

Larger integrated networks are constructed by the union of gene-gene expression correlation information and protein-protein interaction data. Microarray data analysis from studies like obesity, insulin resistance, T2D and kidney failure in diabetes contribute to infer some significant physiological pathways and biological processes related with T2D pathophysiology and complications. The statistical significance analysis of networks has led to the identification of four signature sub-networks that show the interaction across several important metabolic/signalling processes, transcription factors and pathways in T2D. These sub-networks have been further investigated to learn the functional relevance pertaining to disease mechanism which is discussed as follows (Table 1).

**Table 1:** Four top-scored significant networks displaying their corresponding total number of nodes, edges and p-values.

Significant networks	Number of nodes	Number of edges	p-values (Wilcoxon test)
TranscriptionFactors_KidneyComplication	52	146	1.08×10^−5^
*GAPDH*-*EGFR*_MicrovascularComplication	48	162	1.92×10^−7^
Akt/Pi3k pathway_VascularDysfunction	98	357	2.20×10^−10^
Wnt_VascularComplication	49	153	7.39×10^−7^

### GCR Over-Expression Leads to FFA Production, which in Turn Induces c-Fos/c-Jun Activity through the Interactions of TGFBRII

**Figure 2:**
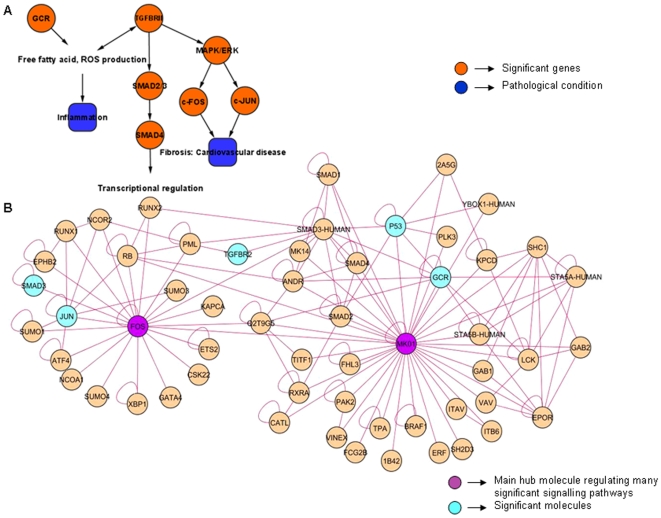
TranscriptionFactors_KidneyComplication network illustrates the interaction of proto-oncogenes with SMAD molecules and nuclear receptors (A-B). All the nodes and edges are in green and purple color respectively. The key molecules which are significantly expressed through their interactions with other molecules in the network are highlighted as orange. The hub molecules are also colored as purple. **A.** Interaction overview (constructed and analyzed in Cytoscape): a skeletal structure of the main sub-network showing only the significant molecules in orange, their biological roles and pathological conditions in blue. **B.** Expanded view of the network imported from Cytoscape: Network explains the interaction of *TGFBRII* and *SMAD3* being modulated by the increased expression of *GCR* (and FFA). This interaction in turn induces expression of early transcription factors which play a role in fibrosis and insulin insensitivity. The main gene hubs are centric to *c-FOS* and *MAPK1* and connected through *SMAD3*.

A signature network (named as TranscriptionFactors_KidneyComplication) obtained from three different datasets viz., Mexican_Hs, Obs_Hs and PCOS_Hs delineates functional relationship between proto-oncogenes, transcription factors, *MAPK* pathway and FFA. The network topology is made of 52 nodes and 146 interactions (p value 1.08×10^−5^). From the given network we identified *c-Fos* and *MAPK1* as hub genes, while *c-Jun* and *GCR* interacted directly with the hubs (Figure 2). *TGFBRII* is shown to link the two hub genes. This network module suggests a relation between FFA, interacting partners of proto-oncogenes like *c-Fos* and *c-Jun*, transcription factor *GCR*, ATP dependent chromatin remodeling factor *SMAD3* and their interaction with *TGFB* and *MAPK* pathways.

*GCR* has been reported to be over-expressed in obesity and cause insulin resistance [62], and is observed in the signature network. Potent up regulation of *GCR* may be considered to represent increased glucocorticoids activity and as inferred from literature, elevated *GC* action is observed in obesity, insulin resistance, T2D and cardiovascular complications. Effect of *GC* includes impaired insulin-dependent glucose uptake in the periphery and enhanced gluconeogenesis in the liver leading to insulin resistance. *GC* and *TGFB* aid in the production of FFA and oxidative stress [63], [64]. This network module shows *TGFB-TGFBRII* interaction causing activation of transcription of several *TGFB* inducible genes via Smad signalling pathway. *SMAD3* is an interacting partner for *TGFBRII* and is up-regulated in the network. Increase in expression of *TGFBR*, *c-Fos* and *c-Jun* is also observed clustered in co-expression gene network. *TGFBR* signalling occurs mainly through the activation of Smad pathway [65]; however it may also involve *MAPK/ERK1/2* pathways in certain cell types, such as endometrial epithelial cells and endometrial stromal cells under certain conditions [66].

Thus the sub network displays activation of growth related proto-oncogenes such as, *c-Fos* and *c-Jun* mediated by *TGFB* and also involving *GCR.*

### Over-Expression of SUMO4 and GAPDH in Response to Oxidative Stress Induces EGFR Signalling to Increase Vascular Complications and Impair Insulin Signalling

**Figure 3:**
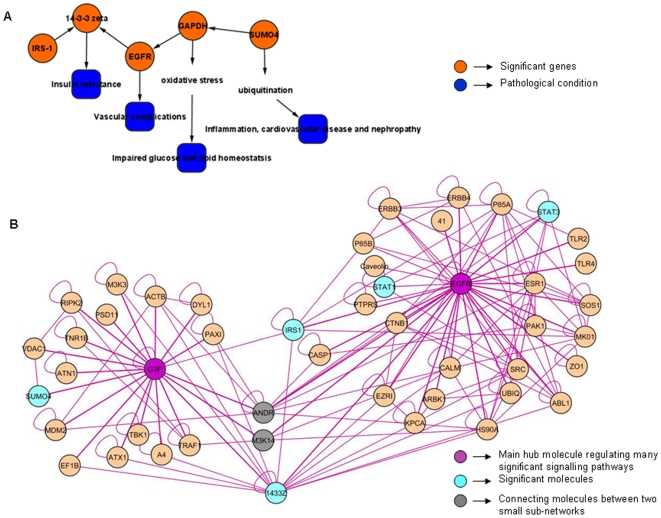
GAPDH-EGFR_MicrovascularComplication network shows EGFR induced insulin resistance and impaired GAPDH induced micro vascular complications (A-B). All the nodes and edges are in green and purple color respectively. The key molecules which are significantly expressed through their interactions with other molecules in the network are highlighted as orange. The molecules connecting two portions of the sub-network are colored as grey. The hub molecules are also colored as purple. **A.** Interaction overview (constructed and analyzed in Cytoscape): a skeletal structure of the main sub-network showing only the significant molecules in orange, their biological roles and pathological conditions in blue. **B.** Expanded view of the network imported from Cytoscape: It shows interaction between stress responsive expression of *GAPDH* with insulin, *SUMO4* and *EGFR*. The *GAPDH* and *EGFR* hubs are interacting directly and are also connected through Androgen receptor, *MAPKKK14* and *14-3-3 protein zeta/delta*. Interaction of *GAPDH* and *EGFR* and subsequent interaction with *14-3-3 zeta* imply their significance in developing insulin insensitivity.

Signature network from Obs_Hs and IR_Hs consists of 48 nodes and 162 interactions (p value 1.92×10^−7^). This network module has been named as GAPDH-EGFR_MicrovascularComplication. It has *GAPDH* and *EGFR* as hub genes. *SUMO4, IRS-1* and *14-3-3 zeta* interact directly with these hub genes. It can be seen that *14-3-3 zeta* links the two hubs (Figure 3).

*SUMO4, GAPDH* and *EGFR* are significantly over expressed in this study and are noted to interact with each other in the network. These interactions have not yet been described for T2D and they appear here from the datasets of T2D and its associated complications. Based on these observations we predicted novel interactions between *SUMO4 and GAPDH*, and *GAPDH and EGFR*. These interactions were further validated using domain and motif information to strengthen the predictions as explained in the subsequent segments.

The interaction between the glycolytic protein *GAPDH* and inflammatory *SUMO4* suggests a potential role in the development of insulin resistance. *EGFR*, an important growth factor receptor, is noted to be up-regulated in diabetic kidney [67], [68]. *EGF* suppresses proteolysis via *PI3-K* in renal tubular cells and increases abundance of *GAPDH* [69]. The interaction of *GAPDH* with *EGFR* as visualized in our network implicates a probable role in insulin signalling. *EGFR* interacts with *14-3-3 zeta* and results in its up regulation as shown in the network. The interaction is mediated through *EGF* to advance *EGFR* signalling [70]. *14-3-3 zeta* interacts with *IRS-1* in this network. In the given network we also observe the up regulation of serine/threonine kinase which participates in *EGF signalling*.

### AKT-1/PI3-K Pathway Is Up-Regulated through Increased Expression of EGFR Eventually Exhibiting Its Two Unique Interactions with PTPN1 and CAV1 in Diabetic Nephropathy

**Figure 4:**
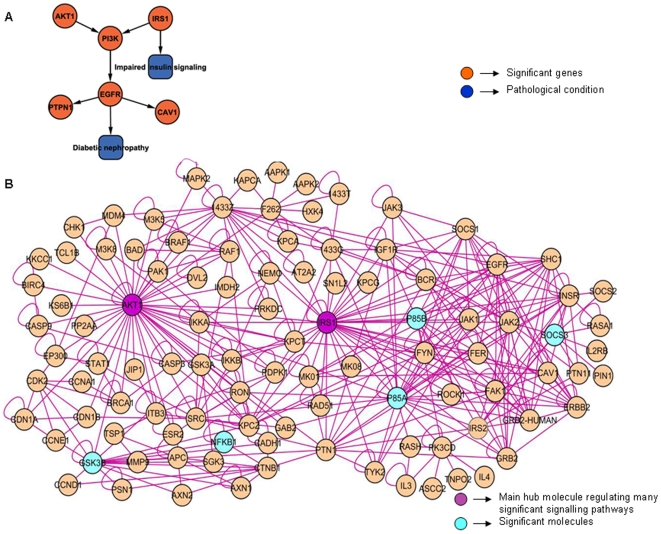
Akt/Pi3k pathway_VascularDysfunction network shows up-regulation of this pathway through increased expression of EGFR leading to diabetic nephropathy and exhibits two unique interactions of EGFR with PTPN1 and CAV1 (A-B). All the nodes and edges are colored as green and purple respectively. The key molecules which are significantly expressed through their interactions with other molecules in the network are highlighted as orange. The hub nodes which have been described here are in purple color. **A.** Interaction Overview (constructed and analyzed in Cytoscape): a skeletal structure of the main sub-network showing only the significant molecules in orange and pathological conditions caused by them in blue. **B.** Expanded view of the network imported from Cytoscape: Different pathways interaction from diabetic nephropathy datasets have been shown here. Deregulated inflammatory cytokines, *AKT1* and *eNOS* involved in kidney dysfunction are shown in this sub-network. Yellow-coloured genes are very significant in this disease pathology.

Sub-network, Akt/Pi3k pathway_VascularDysfunction obtained from diabetes associated with nephropathy datasets namely, DN_Hs and Renalfailure_Mm consists of 98 nodes and 357 interactions (p value 2.20×10^−10^). *AKT1* and *IRS-1* are identified as hub genes which interact with each other directly. Figure 4 displays the causal relationships of *EGFR* with *AKT1*/*PI3-K* pathway in diabetic nephropathy. It also suggests two new interactions between *EGFR*, *PTPN1* and *CAV1* reported for the first time in kidney complications associated with T2D in the network.

*AKT1* activity has been found to be increased in diabetic kidney cells exhibiting its characteristic feature of matrix protein accumulation [71]. High glucose condition is found to induce the *PI3-K*/*AKT* pathway in kidney messengial cells of rodent models mediating insulin signalling through the phosphorylation of *IRS-1*. This subsequently results in over production of collagen I in these cells. Further, the up-regulation of *EGFR* has also been reported as a requirement for *AKT* activation [72]. An interaction between *EGFR* and *PTPN1* has been observed in this network. The occurrence of *PTPN1* is reported in T2D [73]. Protein coded by this gene acts as a key phosphatase for *EGFR*. The phosphatase activity of *PTPN1* is observed to regulate the recruitment of different signals to *EGFR*, which is considered as an important hub molecule in many signalling pathways [74]. Another significant interaction that has been observed here is the interaction between *PTPN1* and *CAV1*. It has been identified as a candidate gene for T2D [75].

On the basis of these observations supported by the literature, we have proposed the interactions of *PTPN1* with *EGFR* and with *CAV1* as the new potential interactions in diabetic nephropathy. Since individually all three molecules are reported to be associated with T2D, it can be suggested that their interactions also might play some significant roles in this disease development.

### Increased Mitochondrial ROS Production Up-Regulates VE-Cadherin Mediated by Wnt Signalling Leading to Vascular Complications

**Figure 5:**
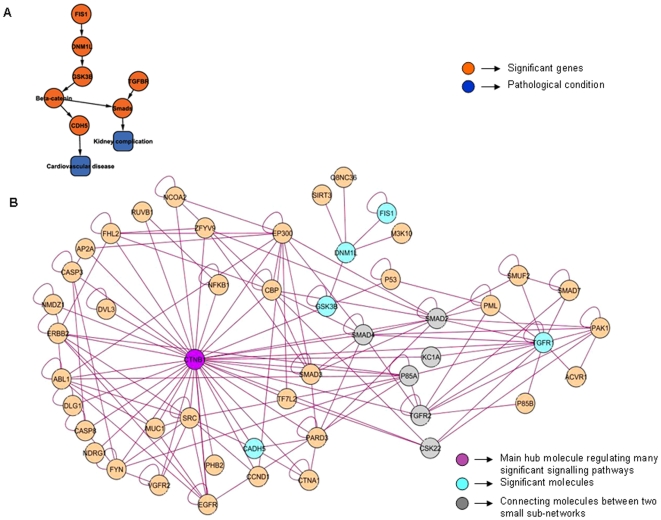
Wnt_VascularComplication network shows the interaction between mitochondrial fission, ROS, Wnt signaling and VE-cadherin causing vascular complication (A-B). All the nodes and edges are in green and purple color respectively. The key molecules which are significantly expressed through their interactions with other molecules in the network are highlighted as orange. The molecules connecting two portions of the sub-network are colored as grey. The hub molecules are also colored as purple. **A.** Interaction overview (constructed and analyzed in Cytoscape): a skeletal view showing only the key interactions where the molecules are shown in orange and the pathological conditions caused by them are in blue. **B.** Expanded view of the network imported from Cytoscape: The *β-catenin* and *TGFB* centric hubs are connected through 6 molecules i.e., *SMAD2 and 4, casein kinases, PIP3K* regulatory subunit alpha and *TGFBR*. *DNM1L* is connected to the *β-catenin* hub through *GSK-3B*. Interaction of *β-catenin* with *VEGFR2* and subsequent interaction with *CDH5* is also illustrated here implying a possible role in kidney complication. It also displays the interaction between *WNK1, SMAD2 and TGFBRII* which is observed only in diabetic nephropathy dataset.

An important signature sub-network noted in three datasets namely, IR_Hs, DN_Hs and Mexican_Hs, shows interaction of pathways causing the generation of ROS, Wnt and TGF-beta pathways. β*-*catenin has been identified as an important hub which interacts with significant genes such as *GSK-3B, CDH5*, *SMAD* and *TGFBRI*. The network, named as Wnt_VascularComplication (Figure 5A) summarizes a network flow of genes involved in these pathways, which further correlates with the significant clinical observations in diabetes subjects. It contains 49 nodes and 153 interactions (p value 7.39×10^−7^).

It has been observed that *DNM1L* and *FIS1* genes are involved in the maintenance of mitochondrial morphology [76], [77]. Illustratively, the proteins produced by *FIS1* and *DNM1L* are noted to be over-expressed and Wnt expression is down-regulated in the networks from expression datasets (Figure 5). *DNM1L* interacts with the molecules of Wnt signalling like *GSK-3B*, thereby regulating its down-stream signalling [78]. Higher expression of *GSK-3B* is thought to cause proteosomal degradation of β-catenin via the formation of a cytoplasmic multiprotein complex [79], [80]. Furthermore, the literature exploration on β-catenin reveals that it gets down-regulated in diabetes nephropathy [81]. In this network β-catenin has been observed to be associated with *CDH5*. It is well established that T2D is often associated with cardiovascular complications where endothelial dysfunction acts as a hallmark. It has been shown that the up-regulation of *CDH5* acts as an indicator of coronary artery disease in patients with diabetes mellitus [82]. Therefore, it is the first instance, where *CDH5* and Wnt are predicted to interact and subsequently lead to vascular complications in diabetes (Figure 5B). Another hub in the network, *TGFBR I* has been recognized as one of the major contributors to diabetic nephropathy mediated by the TGFB-signalling pathway [83]. β-catenin has been found to become associated with TGFB-signalling via its interaction with Smad molecules [84]. These interactions have been noted from the datasets of T2D and diabetic nephropathy as mentioned above. On the basis of these observations we have proposed that these causal relationships might play significant roles in developing kidney complications in diabetes mellitus.

### Assessment of Sub-Networks with Topological Parameters

**Figure 6:**
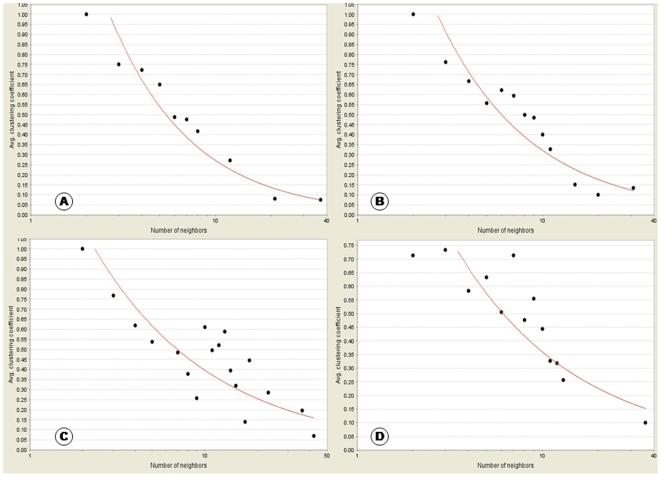
Average clustering coefficient C(k) of all genes with k links follows the scaling law. The average clustering coefficient C(k) is plotted on y-axis as a function of number of neighbours (k) on x-axis for the four networks. The graph exhibits a decreasing tendency of C(k) as k increases. The property follows the power law distribution and shows the nature of scale-free network suggesting a hierarchical organization in the network. (A-D) display the graphical distribution of the four networks namely, (A) TranscriptionFactors_KidneyComplication (R-squared value 0.94). (B) GAPDH-EGFR_MicrovascularComplication (R-squared value 0.857). (C) Akt/Pi3k pathway_VascularDysfunction (R-squared value 0.637). (D) Wnt_VascularComplication (R-squared value 0.787).

Considering the selection criteria of data sets and to identify the altered pathways and gene/protein interactions, we paired our datasets to analyze further. After an exhaustive search of large integrated networks constructed from the selected data sets we identified small putative sub networks. Network topology is thought to render the significance of a node in communicating with other genes or proteins of interest. Parameters like average clustering coefficient, topological coefficient, average degree, power law distribution of degrees and betweenness centrality have been assessed to capture the topology of the networks. Highly cohesive networks have been found to be composed of low-degree nodes (nodes with fewer neighbours) and higher degree nodes are found to have less connected neighbours. The distribution of clustering coefficient is an important characteristic of scale-free networks which decreases as the node degree increases, thereby following a power law distribution. The clustering coefficient of a node is always a number between 0 and 1. The average clustering coefficient characterizes the ‘cliquishness’ of a network. It gives a measurement of average of clustering coefficients for all the nodes with at least two neighbours in a network [85]. The value of average clustering coefficient has been observed to be less in each of the four sub-networks. Average clustering coefficients along with average degrees and network densities are shown in Table 2. In the present analysis the average clustering coefficient and network density for each sub-network appears to be less as compared to the average degree (Table 2). The log-log graph of average clustering coefficient demonstrates that the C(k) decreases as k increases (Figure 6). Figure 6A demonstrates network TranscriptionFactors_KidneyComplication having the best hierarchical scale free organisation. As the number of interactions increase, the average clustering coefficients decline continuously, thereby showing that these data points fit best to the power line curve. The network GAPDH-EGFR_MicrovascularComplication (Figure 6B) also shows a decline in average clustering coefficients, however the decline is not as smooth and gradual as in the first case as some points show more deviations from the power line. In the Wnt_VascularComplication pathway (Figure 6D), initially the average clustering coefficient points are randomly scattered but as the number of neighbours increase the average clustering coefficients begin to decrease. However, in case of the pathway Akt/Pi3k pathway_VascularDysfunction although the average clustering coefficients decrease, they are much more scattered than the rest as shown in Figure 6C. Hence the network is not as scale free as the remaining networks.

**Table 2:** Topological parameters showing the significance of all the four sub-networks.

Four selected sub-networks	Average degree	Network density	Clustering coefficient
TranscriptionFactors_KidneyComplication	4.2	0.07	0.267
*GAPDH*-*EGFR*_MicrovascularComplication	5.25	0.11	0.269
Akt/Pi3k pathway_VascularDysfunction	5.7	0.05	0.242
Wnt_VascularComplication	5.0	0.10	0.247

R-squared value is a statistical measure of the linearity of the curve fit and used to quantify the fit to the power line. It shows the correlation between the given data points and the corresponding points on the fitted power line curve. It gives the proportion of variability in a data set which is explained by a fitted linear model. When the fit is good, the R-squared value is very close to one. The R-squared values for the average clustering coefficient is highest for the network TranscriptionFactors_KidneyComplication (0.94) confirming the observation of best scale free network, followed by GAPDH-EGFR_MicrovascularComplication (0.857), Wnt_VascularComplication pathway (0.787) and Akt/Pi3k pathway_VascularDysfunction (0.631).

**Figure 7:**
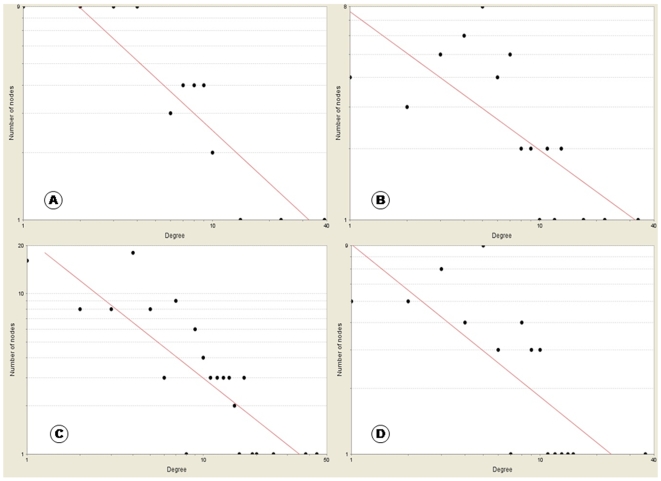
Power law node-degree distribution for the four signature networks. The node degree (k) is represented on the x-axis and the number of nodes with a particular k is represented on the y-axis. The graph displays a decreasing trend of degree distribution with increase in number of links displaying scale free topology. (A-D) display the graphical distribution of the four networks namely, (A) TranscriptionFactors_KidneyComplication (R-squared value 0.778). (B) GAPDH-EGFR_MicrovascularComplication (R-squared value 0.523). (C) Akt/Pi3k pathway_VascularDysfunction (R-squared value 0.695). (D) Wnt_VascularComplication (R-squared value 0.554).

**Figure 8:**
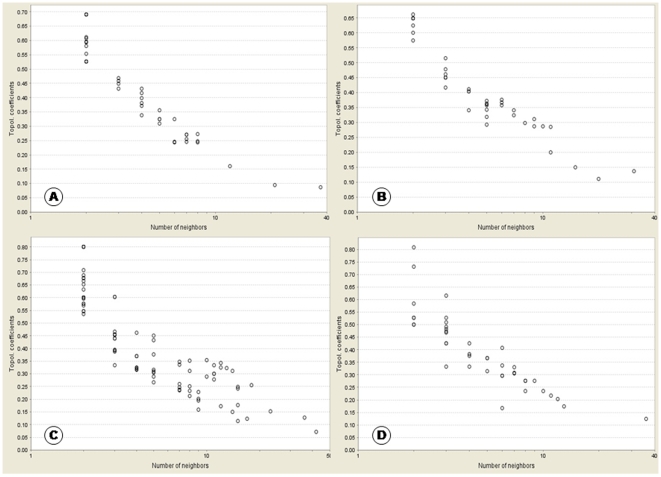
Topological coefficients analysis indicating modular network organization. The distribution of topological coefficient TC (k) is plotted on y-axis and the number of neighbours is plotted on x-axis. The graph shows gradual decrease of this distribution. (A-D) display the graphical distribution of the four networks namely, (A) TranscriptionFactors_KidneyComplication. (B) GAPDH-EGFR_MicrovascularComplication. (C) Akt/Pi3k pathway_VascularDysfunction. (D) Wnt_VascularComplication.

In signalling networks hub nodes are thought to play significant regulatory roles on their adjacent nodes. In such networks the degree distribution P (k) and the topological coefficient TC(k) are expected to be inversely proportional to the number of links. The similar trend is observed in the four networks (Figure 7 and 8) with R-squared values of degree distribution ranging between 0.5–0.8 (Table 3). The networks follow the power law distribution with highest R-squared value of 0.778 for TranscriptionFactors_KidneyComplication exhibiting its strongest distribution. As R*-*squared values approach unity, it implies that the regression approaches a perfect fit. Our results are similar to the general trend with genetic perturbation networks and other gene co-expression networks exhibiting scale free topology (with R-squared values above 0.6–0.7) [86], [87]. The decrease in topological coefficients with the increase in number of neighbours explains that hubs are rather exclusive with rare common neighbours than individual proteins with fewer links. Figure 8D displays that the network Wnt_VascularComplication has one well defined hub. Figures 8A and 8B show that networks TranscriptionFactors_KidneyComplication and GAPDH-EGFR_MicrovascularComplication both have two hubs each, which are connected through few common neighbours. From Figure 8C we observe that the network Akt/Pi3k pathway_VascularDysfunction also has important hubs but there are greater numbers of common neighbours interacting between the main hubs and therefore the hubs are not as distinct as in case of the other networks.

**Table 3:** Value of R-square, degree exponent, threshold value of betweenness centrality and node degree of the four networks.

Networks studied	R-squared value		Value of degree exponent (γ)	Threshold value of betweenness centrality	Threshold value of node degree
	Node degree distribution	Average clustering coefficient			
TranscriptionFactors_KidneyComplication	0.778	0.94	1.296	71.67	4.85
GAPDH-EGFR_MicrovascularComplication	0.523	0.857	1.021	57.25	6.0
Akt/Pi3k pathway_VascularDysfunction	0.695	0.631	1.097	188.51	6.57
Wnt_VascularComplication	0.554	0.787	1.076	68.48	5.62

Table 3 shows the value of γ, the threshold value of betweenness centrality and degree of nodes for the different networks. The value of γ lies between 1 and 2, which is a characteristic of biological networks [88]. The betweenness centrality values are found to be higher than the average value for the hub nodes. The threshold value of betweenness centrality lies between 55 and 190 for the identified networks. The threshold value for node degree for the four networks ranges from 4.85 to 6.57. An over-all scale-free topology is maintained in the four networks. The pattern of network connectivity in these datasets closely resembles a scale-free topology. All the information on network topology provides high confidence to their scale-freeness property supporting the fact that the underlying model is linear. Therefore, it can be suggested that the four networks analysed here are sensitive to the perturbation of more highly connected hubs rather than removal of less connected nodes. There is a scope to obtain further improved results since only the positive data points have been considered for the present analysis.

### Gene Ontology (GO) Verification of Sub-Networks

Similarly functioning proteins tend to form clusters of protein-protein interaction [89]. Sub-networks with overrepresented functional GO categories have been illustrated in pie diagrams (Figure S1A-S1D). The size of each section of the pie chart is basically based on its cluster frequency with statistically significant p-value that allows better visualization of the functional categories. We see in the Figure S1, that most of the molecules are involved in signalling pathways mediated by growth factors, kinases, signal transducer and transcription factors, cytokines and nuclear receptors. Therefore, it can be assumed that the sub-networks represent the clusters of signalling components, which upon alteration may cause T2D and its associated complications.

### Two Novel Interactions and Their Verification

The two novel interactions put forward here are the interaction between *SUMO4* and *GAPDH* and the interaction between *GAPDH* and *EGFR*. Analysis of conserved domains for the two new interactions reveals the following findings. *SUMO4* which is a negative regulator of *NF-kB* interacts with *GAPDH* via its ubiquitin domain. *GAPDH* is a key enzyme in glycolysis and is found to regulate insulin and *EGFR* mediated pathways. Both the components are found to interact in the network obtained from Obs_Hs and IR_Hs. The network GAPDH-EGFR_MicrovascularComplication was identified on the basis of its significance score.

*SUMO4* shows the presence of ubiquitin domain. Ubiquitination is an ATP-dependent process which involves the action of three main enzymes, namely, E1 (ubiquitin activation enzyme), E2 (ubiquitin conjugating enzyme) and E3 (ubiquitin ligase enzyme) [90], [91]. *SUMO4* belongs to the SUMO gene family which encodes small ubiquitin-related modifiers that are attached to proteins and control the target proteins' subcellular localization, stability, or activity.

*GAPDH* participates in energy yielding processes and is found to interact with E2 and E3 enzymatic domains. NO-S-nitrosylation-GAPDH-E3/2 cascade mediates cell death under oxidative stress conditions and thus represent an important mechanism of cytotoxicity [92], [93], [94]. With the help of E3 ligase, ubiquitin is transferred from E2 enzyme to a lysine residue on a substrate protein like *GAPDH* [91].

Ubiquitin-like proteins function as critical regulators of cellular processes and intracellular stress. The C-terminal glycine residue of the ubiquitin-related proteins attach to a lysine side chain of the substrate protein to form an isopeptide linkage [91], [95], [96]. Thus we can hypothesize that protein coded by *SUMO4* interacts with the lysine residues in *GAPDH* and plays a role in regulation of cellular processes such as transcription, signal transduction, repair, autophagy and cell cycle control [97].

The interaction between *GAPDH* and *EGFR* is predicted using the application of InterDom [59], [60], 3did [61] and ELM [98]. We predicted the motifs as well as conserved domains in both *GAPDH* and *EGFR* (as shown in Figure S2 and datasets S3 and S4). Motifs were found out using ELM (Eukaryotic Linear Motif resource). The interactions between domains were predicted using InterDom and the interactions between the chosen motifs and domains was observed using 3did (3D Interacting Domains). The domains and motifs for *SUMO4, GAPDH* and *EGFR* are presented in Datasets S3 and S4. From InterDom, it has been noted that the protein tyrosine kinase domain present in *EGFR* interacts with both C-terminal and N-terminal domains of *GAPDH*. This observation was further validated using 3did, which showed that in the interaction pattern of *GAPDH* and *EGFR*, the protein tyrosine kinase domain of the latter interacts with *GAPDH* motifs via SH2/3_1 domains as illustrated in Figure S2.

## Discussion

Network-based analysis provides system level relationship of molecules across different layers of regulatory controls of biological functions by integrating functional interactions with co-expression information. In order to delineate significant observations, the present study focuses on expression profiles of those interactions/associations, which existed most consistently in maximum number of microarray datasets. The analysis and literature on different biological processes offer an insight into the identification of several facts associated with T2D. Our approach may serve as predictive tool for identifying underlying novel pathways and disease mechanisms in the development of other T2D complications and may also prove useful in providing insight in etiology and progression of other diseases.

A distinct scale-freeness property has been noticed in the four sub-networks as they have decreasing values of coefficients for some topological parameters like average degree, average clustering coefficient and topological coefficient. Simultaneously, betweenness centrality value, another significant topology identifying parameter describes the presence of few important hubs with other nodes. The Wnt_VascularComplication sub-network shows the presence of two distinct hubs, while Akt/Pi3k pathway_VascularDysfunction sub-network displays more hubs with greater number of common neighbours. Betweenness centrality calculates the effectiveness of nodes communicating with other nodes in the sub-network. It is suggested that higher the betweenness value of a node, the higher its significance because a protein with higher degree is more likely to be essential since it has more links. Therefore, threshold betweenness value higher than its average value suggests the importance of the interacting sub-network depending on its nodes' connectivities. All these properties reflect the hierarchical organization and scale-free nature of these sub-networks.

The TranscriptionFactors_KidneyComplication sub-sub-network observed from diabetes, obesity and PCOD datasets suggests a common pathway for causing impaired insulin signalling and generating vascular complications through the interaction between pathways mediated by growth related proto-oncogenes and *GCR*. The sub-network illustrates that increased FFA linked with *GCR* plays a pivotal role in progression of T2D. High amount of FFA has been reported to be associated with diabetes and its complications. FFA is elevated in insulin-resistant subjects because of impaired insulin-dependent down-regulation of lipolysis. Increased FFAs competitively inhibit oxidation of glucose, contributing to the development of insulin resistance [63] and also affect ROS generation thereby acting as source of oxidative stress [99]. *GC* may increase circulating FFA by inhibiting lipoprotein lipase and thereby increase both the uptake and turnover of fatty acids in adipose tissue. Effect of *GC* includes impaired insulin-dependent glucose uptake in the periphery and enhanced gluconeogenesis in the liver leading to insulin resistance [63], [64]. The TranscriptionFactors_KidneyComplication sub-network reveals up regulation of *GCR*. Increased *GCR* sensitivity can be considered as an indicator of excessive activity of *GC* which is a plausible contributor to obesity and insulin resistance. FFA induces the over-expression of *TGFBRII* as noted in diabetic kidney disease [100] and it increases the expressions of proto-oncogenes and *SMAD3* in TGFB signalling process [101]. *TGFB* induces as well as gets activated by ROS intermediates [102], [65]. ROS stimulates early growth signals including induction of *c-Fos* and *c-Myc* mRNA expression via TGFB signalling [103]. Growth related proto-oncogenes exhibit high expression in early phase of glomerular hypertrophy during hyperglycemia [104], [105]. Literature also reports that systemic short chain fatty acids can up regulate the expression of early response genes such as *c-Myc, c-Fos* and *c-Jun* [106]. These genes can bind to the (AP)-1 sites of the promoters of their target genes like *fibronectin* and result in their differential transcription [66]. Moreover, the over-expression of *TGFBRII* during high glucose concentration leads to an increase in expression of *type 1 collagen* and accumulation of extra cellular matrix [107]. Excessive collagen and matrix proteins deposition is characteristic of fibrosis [108], [109]. We hypothesize that such an association involving FFA, *TGFB-TGFBRII* and proto-oncogenes could eventually lead to fibrosis of renal cells when glucose concentration is high. This sub-network shows the existence of considerable number of genes playing roles in signal transduction, positive regulation of transcription and regulation of cell cycle through gene ontology analysis. It also displays the involvement of genes in molecular functions like protein serine/threonine kinase activity and *TGFBR* activity.

Sub-network from obesity and diabetes datasets indicate the significant roles of *SUMO4*, *GAPDH* and *EGFR* interactions in insulin signalling diabetes progression. From the information gathered regarding their individual functions, we propose that under conditions of stress and obesity, the interactions between *SUMO4* and *GAPDH* play a role in regulating insulin and *EGFR* signalling to increase vascular complications in diabetic subjects. In diabetes, ubiquitin/proteasome over activity is associated with enhanced inflammatory activity induced by oxidative stress. In response to FFA, *SUMO4* is capable of inducing the expression of inflammatory cytokines which target vessels and kidney in cardiovascular and renal diseases. The variants of *SUMO4* have been reported to be associated with T2D and diabetic nephropathy via the induction of *NF-kB* pathway. *SUMO4* gene encodes small ubiquitin like modifier 4, which alters immune response through *IkBa*, and regulates *NF-kB* activation [110], [111]. *GAPDH* over-expression may be attributed to compensate for the progressive decrease in muscle mitochondrial function due to FFA induced ROS and contribute to loss of glucose and lipid homeostasis and eventually obesity and T2D [112], [113]. *GAPDH* can mediate cell death associated with oxidative stress [92]. Our sub-network displays *SUMO4* and *EGFR* interacting with *GAPDH*. Interaction between *GAPDH* and *SUMO4* suggests that they may be interacting together through ubiquitination process. It is inferred from literature that *GAPDH* (*lys*) can interact with ubiquitin ligase enzyme (E3) and form an isopeptide bond with the C-terminal glycine motifs of *SUMO4* [91], [93]. In the interaction pattern of *GAPDH* and *EGFR*, the protein tyrosine kinase domain of the latter has been predicted to interact with *GAPDH*. Therefore, it can be envisaged that *GAPDH* is getting phosphorylated by *EGFR* tyrosine kinase. These interactions were verified using 3did and Interdom (Figure S2 and Datasets S3 and S4) and suggest a role in the progression of T2D accompanied with obesity. GAPDH-EGFR_MicrovascularComplication sub-network further displays interaction between *EGFR* and *14-3-3 zeta*. Up regulation of *EGFR* has been reported to cause vascular complications in diabetic rodent models [114], [115]. Proteins belonging to the *14-3-3* family are involved in metabolism, cell survival and proliferation [116]. Association of *IRS-1* with *14-3-3* protein is reported to play a role in the regulation of insulin sensitivity by interrupting the interaction between the insulin receptor and *IRS* [117]. Thus we deduce that *EGFR* can interact with *IRS-1* via *14-3-3 zeta* and result in impaired insulin signalling. It has been reported that agents such as FFAs, cytokines, cellular stress and hyperinsulinemia, induce insulin resistance, and lead to activation of several serine/threonine kinases and phosphorylation of *IRS-1* as well. These agents negatively regulate *IRS-1* functions by phosphorylation [118]. Gene ontology analysis of this sub-network establishes its enrichment with signal transduction, phosphorylation and signalling pathway mediated by insulin receptor. Molecular functions like protein kinase and *MAPK* activities and *EGFR* activity have been found to be associated with significant number of genes from the sub-network.

Sub-network from diabetic nephropathy datasets exhibits increased expression of *AKT1* which gets accumulated in diabetic kidneys. This gene mediates insulin signalling by inducing *PI3-K*. On the other hand, up-regulation of EGFR has been observed to activate *AKT1* [72]. Protein coded by PTPN1, a tyrosine phosphatase kinase gene is a negative regulator of insulin signalling and has been reported to be associated with diabetes mellitus [119], [73]. This gene has been identified as a potential drug target for treating obesity and T2D [120]. *PTPN1* has been observed to interact with *EGFR* in the present sub-network. Signalling pathway mediated by *EGFR* plays central role in regulating numerous other signalling pathways. The phosphatise activity of *PTPN1* has been shown to regulate many incoming and outgoing signals to *EGFR* [74]. Subsequently, the interplay between *PTPN1* and *CAV1* has been noted here. Studies show that *PTPN1* has a binding site for *CAV1*. The association has been found to modulate the activity of *PTPN1* [121]. Up-regulation of *CAV1* has been found to contribute to the development of T2D [75]. Protein coded by *CAV1* is known to be involved in signal transduction and many cellular processes. It has been observed that the scaffolding domain of the protein coded by *CAV1* binds directly to the insulin receptor thereby regulating glucose homeostasis [122]. Analysis of gene ontology exhibits the gene abundance, which play a significant role in processes such as insulin receptor signalling, cytoskeletal protein binding. Considerable numbers of genes participate in molecular functions like protein tyrosine kinase activity and *EGFR* activity. From this diabetic nephropathy sub-network module we have proposed the two putative interactions of *PTPN1* with *EGFR* and *CAV1* which have been highlighted for the first time in diabetes condition.

A common trend across the sub-network Wnt_VascularComplication describes the significance of oxidative stress modulating *Wnt/β-catenin* and *CDH5* in diabetes. Oxidative stress produced by the overproduction of ROS depends on mitochondrial morphology and plays a major role in beta-cell dysfunction, insulin resistance, glucose intolerance and above all, T2D [76], [123]. Genes involved in oxidative stress like *FIS1* and *DNM1L* are shown to modulate Wnt signalling via *GSK-3B*. Both *DNM1L* and *FIS1* are involved in the maintenance of mitochondrial morphology, which regulates molecules of Wnt signalling pathway like β-catenin and *GSK-3B* [124], [125]. It exists in two isoforms i.e., alpha (*GSK-3A*) and beta (*GSK-3B*) which are coded by two separate genes. Elevated level of *GSK-3B* has been observed to contribute to diabetes development. Targeting and synthesizing selective inhibitors of this molecule have been shown to modulate insulin sensitivity [126].This gene eventually has been reported to interact with β-catenin [78]. β-catenin serves as a substrate of GSK-3B by phosphorylation. Over-expression of GSK-3B increases the phosphorylation of β-catenin resulting in the formation of a cytoplasmic multi-protein complex. This induces the degradation of β-catenin in a proteosomal pathway [77], [78]. The association between *CDH5* and β-catenin suggests their involvement in the pathogenesis of T2D. The interactions of β-catenin with *CDH5* and *TGFBRI* have been noted from exclusively diabetes mellitus dataset and also from diabetic nephropathy dataset. The protein coded by *CDH5* has been specifically found to express in vascular endothelial cells. β-catenin has been found to interact with *CDH5* in cell adhesion. It is strongly suggested that signalling mediated by β-catenin modulates the organization and function of endothelial cells. Degradation of this protein might impair the growth of endothelial cells [127]. Therefore, we propose that the association of *CDH5* with β-catenin might play a significant role in diabetes mellitus through the impairment of vascular endothelial cell function. Gene ontology analysis of this sub-network module shows majority of genes belonging to the biological processes like signal transduction, phosphorylation and positive regulation of transcription. Molecular functions such as signal transducer activity and protein kinase cascade activity has also been related with several genes from the sub-network. An intensive biochemical analysis on these interactions can bring more insight into the understanding of their causal relationships in T2D. Therefore, there is a wide scope to analyze further these interactions which could probably render into the development of new gene/protein targets eventually leading to the development of therapeutic drugs.

## Supporting Information

Figure S1 Over-representation of gene ontology categories from the four selected sub-networks (A-D): The enrichment of significant GO terms (biological processes and molecular functions) with the genes present in the networks. Each GO category has been calculated using the percent frequency of that category enriched with nodes. (A). Illustrates the GO categories for the network TranscriptionFactors_KidneyComplication showing the significance of signal transduction, regulation of cell cycle and positive regulation of transcription. (B). The network GAPDH-EGFR_MicrovascularComplication showing greater enrichment of categories signal transduction and phosphorylation than the others. (C). Exhibits the major distribution of biological processes like apoptosis and insulin receptor signalling pathway from the network Akt/Pi3k pathway_VascularDysfunction. (D). Shows the distribution of GO categories for the sub-network Wnt_VascularComplication. The categories of signal transduction, phosphorylation and positive regulation of transcription show maximum enrichment suggesting that majority of genes participate in signalling pathways and phosphorylation processes.
(0.78 MB TIF)

Figure S2 Interaction between EGFR and GAPDH through Protein Tyrosine Kinase domain of EGFR and motifs of GAPDH: Protein Tyrosine Kinase domain of EGFR interacts with C-terminal and N-terminal domains of GAPDH. Information on motifs for the genes, was got using Eukaryotic Linear Motif resource (ELM). The interaction between the motifs (LIG_SH2_SRC, LIG_SH2_STAT5 and LIG_SH3_3) of GAPDH and EGFR can be visualised using 3did. The interaction with Protein Tyrosine Kinase domain takes place via SH2/3_1 domains.
(1.02 MB TIF)

Dataset S1 Details of the datasets on their tissue source, control sets and diseased sets.
(0.03 MB DOC)

Dataset S2 Statistically determined up-regulated and down-regulated genes from microarray studies.
(0.03 MB DOC)

Dataset S3 Interacting domains for SUMO4, GAPDH and EGFR.
(0.03 MB DOC)

Dataset S4 List of interacting motifs for SUMO4, GAPDH and EGFR.
(0.07 MB DOC)
