# Supplementary figures and images for: Expression-Based Network Biology Identifies Alteration in Key Regulatory Pathways of Type 2 Diabetes and Associated Risk/Complications
Source: PLoS One. 2009 Dec 7;4(12):e8100. doi: 10.1371/journal.pone.0008100 (PMC2785475; doi:10.1371/journal.pone.0008100)

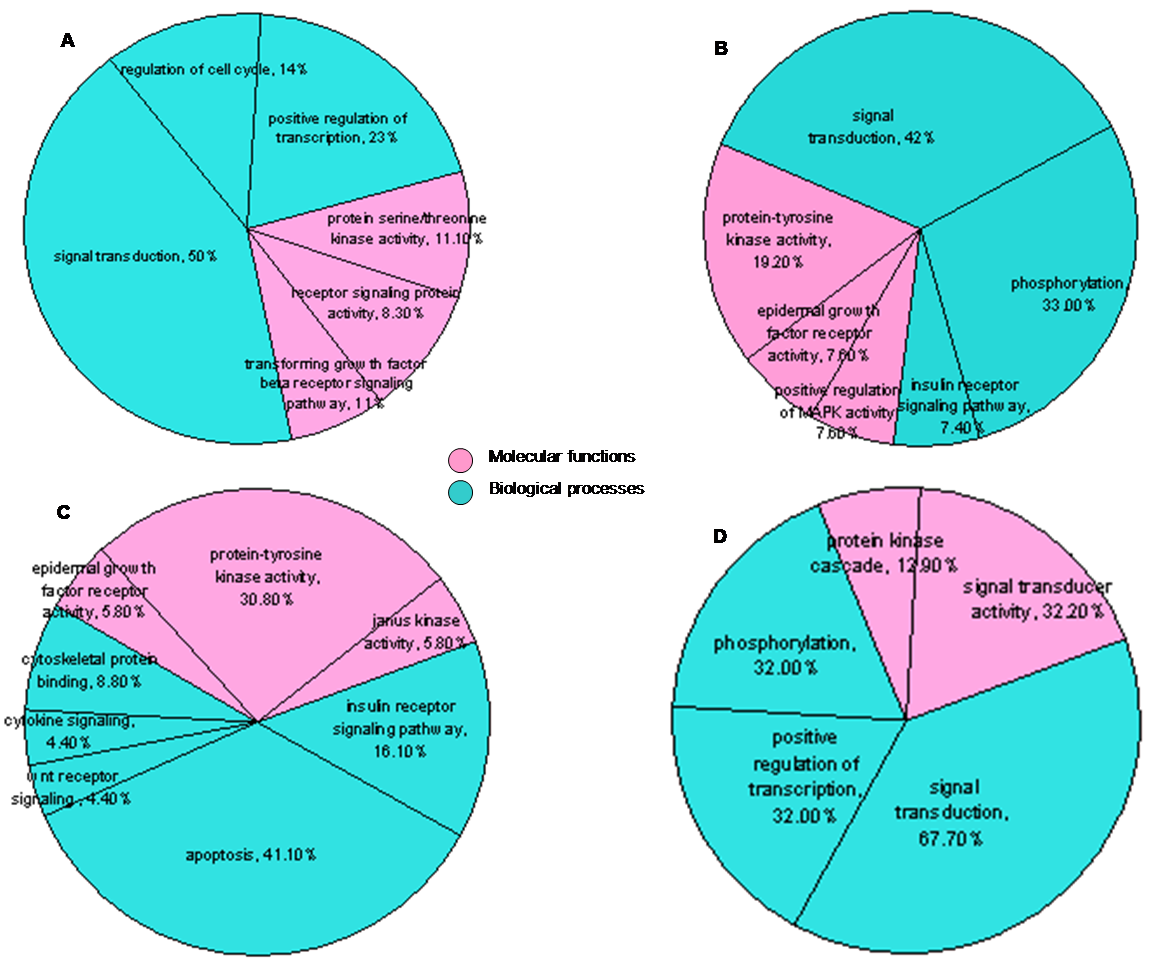

Supplement: Figure S1 — Over-representation of gene ontology categories from the four selected sub-networks (A-D): The enrichment of significant GO terms (biological processes and molecular functions) with the genes present in the networks. Each GO category has been calculated using the percent frequency of that category enriched with nodes. (A). Illustrates the GO categories for the network TranscriptionFactors_KidneyComplication showing the significance of signal transduction, regulation of cell cycle and positive regulation of transcription. (B). The network GAPDH-EGFR_MicrovascularComplication showing greater enrichment of categories signal transduction and phosphorylation than the others. (C). Exhibits the major distribution of biological processes like apoptosis and insulin receptor signalling pathway from the network Akt/Pi3k pathway_VascularDysfunction. (D). Shows the distribution of GO categories for the sub-network Wnt_VascularComplication. The categories of signal transduction, phosphorylation and positive regulation of transcription show maximum enrichment suggesting that majority of genes participate in signalling pathways and phosphorylation processes. (0.78 MB TIF) [file pone.0008100.s001.tif]

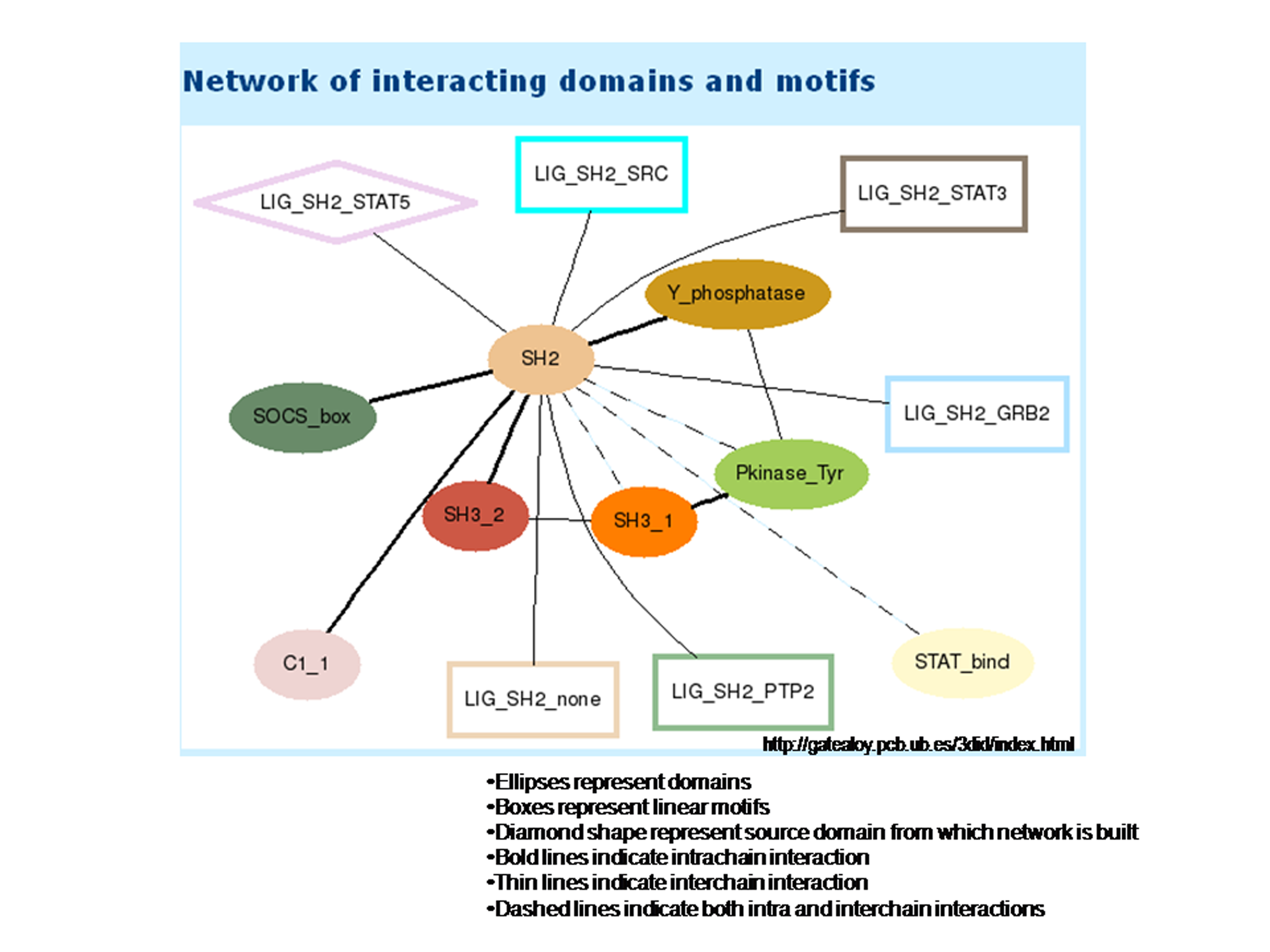

Supplement: Figure S2 — Interaction between EGFR and GAPDH through Protein Tyrosine Kinase domain of EGFR and motifs of GAPDH: Protein Tyrosine Kinase domain of EGFR interacts with C-terminal and N-terminal domains of GAPDH. Information on motifs for the genes, was got using Eukaryotic Linear Motif resource (ELM). The interaction between the motifs (LIG_SH2_SRC, LIG_SH2_STAT5 and LIG_SH3_3) of GAPDH and EGFR can be visualised using 3did. The interaction with Protein Tyrosine Kinase domain takes place via SH2/3_1 domains. (1.02 MB TIF) [file pone.0008100.s002.tif]
