# Supplementary material for: Expression-Based Network Biology Identifies Alteration in Key Regulatory Pathways of Type 2 Diabetes and Associated Risk/Complications
Source: PLoS One. 2009 Dec 7;4(12):e8100. doi: 10.1371/journal.pone.0008100 (PMC2785475; doi:10.1371/journal.pone.0008100)
Supplement: Dataset S1 — Details of the datasets on their tissue source, control sets and diseased sets. (0.03 MB DOC) [file pone.0008100.s003.doc]

| **Dataset** | **Tissue source** | **Control sets** | **Diseased sets** |
| --- | --- | --- | --- |
| IR_Hs | Vastus lateralis muscle | 5 (insulin sensitive) | 5 (insulin resistant) |
| Preadipocyte_Hs | Abdominal subcutaneous preadipocytes | 14 (lean) | 14 (obese) |
| Adipocyte_Hs | Abdominal subcutaneous adipocytes | 20 (lean) | 19 (obese) |
| Obs_Hs | Skeletal muscle | 8 (non obese) | 8 (obese)  8 (morbidly obese) |
| PCOS_Hs | Adipose tissue | 7 (normal) | 8 (polycystic ovary  syndrome) |
| DN_Hs | Glomeruli from kidney | 3 (normal) | 3 (diabetic  nephropathy) |
| Mexican_Hs | Skeletal muscle | 10 (with either positive or  negative family history of  diabetes) | 6 (T2D patients) |
