# Supplementary material for: Expression-Based Network Biology Identifies Alteration in Key Regulatory Pathways of Type 2 Diabetes and Associated Risk/Complications
Source: PLoS One. 2009 Dec 7;4(12):e8100. doi: 10.1371/journal.pone.0008100 (PMC2785475; doi:10.1371/journal.pone.0008100)
Supplement: Dataset S2 — Statistically determined up-regulated and down-regulated genes from microarray studies. (0.03 MB DOC) [file pone.0008100.s004.doc]

| **Datasets studied** | **Up-regulated genes** | **Down-regulated genes** |
| --- | --- | --- |
| IR-Hs | 264 | 238 |
| Preadipocyte_Hs | 140 | 139 |
| Adipocyte_Hs | 236 | 322 |
| Obs_Hs | 308 | 165 |
| PCOS_Hs | 69 | 215 |
| DN_Hs | 603 | 289 |
| Mexican_Hs | 167 | 128 |
| Renalfailure_Mm | 142 | 131 |
