# Supplementary material for: Expression-Based Network Biology Identifies Alteration in Key Regulatory Pathways of Type 2 Diabetes and Associated Risk/Complications
Source: PLoS One. 2009 Dec 7;4(12):e8100. doi: 10.1371/journal.pone.0008100 (PMC2785475; doi:10.1371/journal.pone.0008100)
Supplement: Dataset S3 — Interacting domains for SUMO4, GAPDH and EGFR. (0.03 MB DOC) [file pone.0008100.s005.doc]

| **SUMO4** | **GAPDH** | **EGFR** |
| --- | --- | --- |
| N terminal ubiquitin-like domain | NAD binding domain | Furin-like cysteine rich |
|  | C terminal domain | Receptor L domain |
|  | Dihydrodipicolinate reductase, N terminus | Protein Tyrosine Kinase |
|  |  | YLP motif |
