# Supplementary material for: Expression-Based Network Biology Identifies Alteration in Key Regulatory Pathways of Type 2 Diabetes and Associated Risk/Complications
Source: PLoS One. 2009 Dec 7;4(12):e8100. doi: 10.1371/journal.pone.0008100 (PMC2785475; doi:10.1371/journal.pone.0008100)
Supplement: Dataset S4 — List of interacting motifs for SUMO4, GAPDH and EGFR. (0.07 MB DOC) [file pone.0008100.s006.doc]

| **SUMO4** | **GAPDH** | **EGFR** |
| --- | --- | --- |
| [CLV_PCSK_PC1ET2_1](http://elm.eu.org/elmPages/CLV_PCSK_PC1ET2_1.html) | [CLV_PCSK_SKI1_1](http://elm.eu.org/elmPages/CLV_PCSK_SKI1_1.html) | CLV_NDR_NDR_1 |
| [LIG_CYCLIN_1](http://elm.eu.org/elmPages/LIG_CYCLIN_1.html) | [LIG_AP2alpha_2](http://elm.eu.org/elmPages/LIG_AP2alpha_2.html) | CLV_PCSK_PC1ET2_1 |
| [LIG_FHA_1](http://elm.eu.org/elmPages/LIG_FHA_1.html) | [LIG_APCC_Dbox_1](http://elm.eu.org/elmPages/LIG_APCC_Dbox_1.html) | CLV_PCSK_SKI1_1 |
| [LIG_PDZ_3](http://elm.eu.org/elmPages/LIG_PDZ_3.html) | [LIG_BRCT_BRCA1_1](http://elm.eu.org/elmPages/LIG_BRCT_BRCA1_1.html) | LIG_14-3-3_1 |
| [LIG_PP1](http://elm.eu.org/elmPages/LIG_PP1.html) | [LIG_FHA_1](http://elm.eu.org/elmPages/LIG_FHA_1.html) | LIG_APCC_Dbox_1 |
| [LIG_SH2_STAT5](http://elm.eu.org/elmPages/LIG_SH2_STAT5.html) | [LIG_FHA_2](http://elm.eu.org/elmPages/LIG_FHA_2.html) | LIG_BRCT_BRCA1_1 |
| [LIG_TRAF2_1](http://elm.eu.org/elmPages/LIG_TRAF2_1.html) | [LIG_PDZ_3](http://elm.eu.org/elmPages/LIG_PDZ_3.html) | LIG_CYCLIN_1 |
| [LIG_WW_4](http://elm.eu.org/elmPages/LIG_WW_4.html) | [LIG_SH2_GRB2](http://elm.eu.org/elmPages/LIG_SH2_GRB2.html) | LIG_FHA_1 |
| [MOD_GlcNHglycan](http://elm.eu.org/elmPages/MOD_GlcNHglycan.html) | [LIG_SH2_SRC](http://elm.eu.org/elmPages/LIG_SH2_SRC.html) | LIG_FHA_2 |
| [MOD_PKA_1](http://elm.eu.org/elmPages/MOD_PKA_1.html) | [LIG_SH2_STAT5](http://elm.eu.org/elmPages/LIG_SH2_STAT5.html) | LIG_MAPK_1 |
| [MOD_PKA_2](http://elm.eu.org/elmPages/MOD_PKA_2.html) | [LIG_SH3_3](http://elm.eu.org/elmPages/LIG_SH3_3.html) | LIG_NRBOX |
| [MOD_PLK](http://elm.eu.org/elmPages/MOD_PLK.html) | [LIG_USP7_1](http://elm.eu.org/elmPages/LIG_USP7_1.html) | LIG_PDZ_3 |
| [MOD_ProDKin_1](http://elm.eu.org/elmPages/MOD_ProDKin_1.html) | [MOD_CK1_1](http://elm.eu.org/elmPages/MOD_CK1_1.html) | LIG_PTB_1 |
| [MOD_SUMO](http://elm.eu.org/elmPages/MOD_SUMO.html) | [MOD_CK2_1](http://elm.eu.org/elmPages/MOD_CK2_1.html) | LIG_PTB_2 |
|  | [MOD_GlcNHglycan](http://elm.eu.org/elmPages/MOD_GlcNHglycan.html) | LIG_RGD |
|  | [MOD_GSK3_1](http://elm.eu.org/elmPages/MOD_GSK3_1.html) | LIG_SH2_GRB2 |
|  | [MOD_N-GLC_1](http://elm.eu.org/elmPages/MOD_N-GLC_1.html) | LIG_SH2_SRC |
|  | [MOD_PIKK_1](http://elm.eu.org/elmPages/MOD_PIKK_1.html) | LIG_SH2_STAT3 |
|  | [MOD_PLK](http://elm.eu.org/elmPages/MOD_PLK.html) | LIG_SH2_STAT5 |
|  | [MOD_SUMO](http://elm.eu.org/elmPages/MOD_SUMO.html) | LIG_SH3_1 |
|  | [TRG_LysEnd_APsAcLL_1](http://elm.eu.org/elmPages/TRG_LysEnd_APsAcLL_1.html) | LIG_SH3_3 |
|  |  | LIG_TRAF2_1 |
|  |  | LIG_TRAF6 |
|  |  | LIG_USP7_1 |
|  |  | LIG_WW_4 |
|  |  | MOD_CK1_1 |
|  |  | MOD_CK2_1 |
|  |  | MOD_GlcNHglycan |
|  |  | MOD_GSK3_1 |
|  |  | MOD_N-GLC_1 |
|  |  | MOD_N-GLC_2 |
|  |  | MOD_OFUCOSY |
|  |  | MOD_PIKK_1 |
|  |  | MOD_PKA_1 |
|  |  | MOD_PKA_2 |
|  |  | MOD_PKB_1 |
|  |  | MOD_PLK |
|  |  | TRG_ENDOCYTIC_2 |
|  |  | TRG_LysEnd_APsAcLL_1 |
|  |  | TRG_PEX |
